# Effects of Bile Acid Supplementation on Lactation Performance, Nutrient Intake, Antioxidative Status, and Serum Biochemistry in Mid-Lactation Dairy Cows

**DOI:** 10.3390/ani14020290

**Published:** 2024-01-17

**Authors:** Yuhang Chen, Cong Yuan, Tianyu Yang, Han Song, Kang Zhan, Guoqi Zhao

**Affiliations:** 1 Institute of Animal Culture Collection and Application, College of Animal Science and Technology, Yangzhou University, Yangzhou 225009, China; chenyuhang940421@163.com (Y.C.); yuancongyzu@163.com (C.Y.); 18762304846@163.com (T.Y.); 13273815249@163.com (H.S.); kzhan@yzu.edu.cn (K.Z.); 2 Institutes of Agricultural Science and Technology Development, Yangzhou University, Yangzhou 225009, China; 3 Joint International Research Laboratory of Agriculture and Agri-Product Safety, The Ministry of Education of China, Yangzhou University, Yangzhou 225009, China

**Keywords:** bile acid, mid-lactation dairy cows, antioxidant

## Abstract

**Simple Summary:**

Bile acids (BAs) improve antioxidant properties and lipid metabolism, but their effects on dairy cattle remain to be elucidated. The study results revealed that total antioxidant capacity and levels of superoxide dismutase and glutathione peroxidase were significantly higher in dairy cows supplemented with 18 g of BAs than in control cows. In conclusion, BAs can improve the antioxidant performance of dairy cows.

**Abstract:**

This experiment investigated the effects of different levels of bile acid (BA) additives in diets on the lactation performance, serum antioxidant metabolites, and serum biochemical indices of 60 multiparous mid-lactation dairy cows. The cows were randomized to receive one of the four homogeneous treatments, with the BA preparation supplemented at 0, 6, 12, and 18 g/head/d. The experiment lasted for 14 weeks. The first 2 weeks were the pre-feeding period. The milk yield and composition data were recorded weekly, and the dry matter intake and antioxidative blood index were analyzed on the 6th, 10th, and 14th weeks of the study. On the 84th day of the experiment, the experimental group exhibited significantly higher levels of total protein and albumin, by 57.5% and 55.6%, respectively, compared to the control group (*p* < 0.05). On both the 28th and 84th days of the trial, the experimental group showed a markedly higher lipase content compared to the control group, by 26.5% and 25.2%, respectively (*p* < 0.05). Furthermore, the experimental group displayed notably elevated levels of superoxide dismutase, glutathione peroxidase, and total antioxidant capacity, surpassing the control group by 17.4%, 21.6%, and 8.7%, respectively. In conclusion, BA additives improve the serum antioxidant indices of dairy cows, thereby enhancing the performance of these cows.

## 1. Introduction

Reactive oxygen species (ROS) are a group of chemical substances produced during normal cellular respiration. They include superoxide anion radical (O^2^), hydrogen peroxide (H_2_O_2_), hydroxyl radical (OH–), and others. ROS play important physiological roles within cells, including serving as essential components of cellular energy metabolism to provide the required energy for maintaining normal cellular activities [1,2].

However, excessive production of ROS can cause damage to cells. To maintain the redox balance within cells, cells possess a sophisticated antioxidant defense system to eliminate excess ROS. This antioxidant defense system includes enzymes such as superoxide dismutase (SOD), glutathione peroxidase (GPX), and glutathione reductase (GR), as well as small molecules like glutathione (GSH) [2,3]. These defense systems function to effectively neutralize ROS and maintain the redox balance within cells.

However, when the production of ROS exceeds the clearance capacity of the antioxidant defense system, oxidative stress occurs. Oxidative stress refers to the imbalance between ROS and the cellular antioxidant defense system, leading to abnormal changes in cell structure, function, and metabolism. Excessive production and accumulation of ROS can cause lipid peroxidation, protein oxidation, DNA damage, and other harmful effects on cells and tissues [4]. In animals, excessive ROS production is closely associated with the occurrence and development of many metabolic diseases, such as diabetes and obesity [4]. Therefore, enhancing antioxidant status to address oxidative stress-related issues has become an important research direction. During lactation in dairy cows, the mammary gland undergoes the rapid development and synthesis of large amounts of milk components, which requires a significant energy supply. However, this process is often accompanied by oxidative stress [5]. Studies have shown that during lactation, cows experience a decrease in energy metabolism, reduced antioxidant enzyme activity, hormonal changes, and other factors that lead to a decline in their antioxidant capacity [6]. Therefore, improving the antioxidant capacity of cows during lactation is of great significance for maintaining their health status and milk quality [6,7,8]. In recent years, there has been a growing awareness that adding functional supplements to the feed can effectively improve the health status of dairy cows. These functional supplements are typically natural bioactive substances extracted from animals or plants. Due to their simple biodegradability and relatively high safety, they are widely regarded as important additives. These natural bioactive substances include polyphenolic compounds, antioxidant enzymes, vitamins, etc., which possess strong antioxidant abilities. They can neutralize excess ROS and alleviate the severity of oxidative stress [9,10,11].

Bile acids (BAs) are the main components of bile, synthesized by liver cells and excreted into bile. They are widely present in bile and the small intestine. Bile acids flow into the small intestine with bile and play an important role in the digestion and absorption of fats. Bile acids are endogenous molecules synthesized from cholesterol in vertebrates [12] and are considered versatile mediators that regulate various physiological processes and the utilization of different nutrients. Due to their amphipathic structure, bile acids can form micelles to solubilize lipids, facilitating the digestion of fats, cholesterol, and fat-soluble vitamins [13]. Bile acids participate in the regulation of antioxidant defense through multiple mechanisms, including scavenging free radicals, promoting antioxidant enzyme activity, regulating mitochondrial function, and modulating the expression of antioxidant genes. These regulatory effects help maintain the oxidative–antioxidative balance in cells and tissues, protecting them from oxidative stress damage [14,15]. Studies have reported that the addition of BAs to the diet of broiler chickens enhances their antioxidant capacity, increases the oxygen radical absorbance capacity in serum, and reduces the levels of malondialdehyde [16]. Furthermore, supplementing BAs significantly increases the lysozyme activity and immunoglobulin content in the intestine of *Cynoglossus semiliaevis*, thereby significantly improving intestinal antioxidant capacity by increasing antioxidant enzyme activity and reducing malondialdehyde levels [17]. It has been reported that bile acids are synthesized from cholesterol, and through the excretion of bile acids, livestock animals can eliminate excess cholesterol, thus maintaining cholesterol balance, which is crucial for preventing lipid metabolism disorders and cholesterol deposition.

However, there is limited research on the effects of BAs on mid-lactation cows. Therefore, the aim of this study is to evaluate the effects of dietary supplementation of BAs on the lactation performance, antioxidant capacity, and blood metabolites of mid-lactation cows.

## 2. Materials and Methods

### 2.1. Animal Ethical Statement

All Holstein cows used in this study were strictly cared for as per the principles of the Institutional Animal Care and Use Committee (IACUC) of Yangzhou University (SYXK (Su) 2016-0019).

### 2.2. Preparation for BAs

The preparation process of the BAs simply consisted of the following five steps: saponification, decolorization, acidification, purification, and recrystallization of the porcine gall bladders to obtain the pure bile acid (Shandong Longchang Animal Health Care Co., Ltd., Dezhou, China). All the BAs used in the test were prepared by Shandong Longchang Animal Health Care Co., Ltd., (Dezhou, China); BA concentration was >95.0%. Bile salts were gently mixed with a portion of the basal diet and then mixed with all the experimental diets. The composition of bile salts was 96%, hyodeoxycholic acid was 78%, and chenodeoxycholic acid was 18.4%. The amount of each BA in this product was determined using high-performance liquid chromatography [18].

### 2.3. Experimental Design and Diets

The experiment was conducted from February to June 2022 at the experimental base of the Animal Nutrition and Feed Engineering Research Center of Yangzhou University (China). Sixty healthy, lactating, Holstein cows having a similar body condition (612 ± 11 kg), parity (second), and days in milk (DIM) (136.96 ± 0.79 day) were selected and randomly divided into four groups: (1) control group fed total mixed ration (TMR) (CON group, *n* = 15); (2) 6/g/d (BAs group, *n* = 15), 12/g/d (BAs group, *n* = 15), and 18/g/d (BAs group, *n* = 15). The dietary ingredients and nutrition composition were formulated to meet NRC (2001) recommendations and previously described nutritional requirements (Table 1). The cows were fed with the whole mixed diet three times a day at 8:00, 14:00, and 21:00, respectively. Milking coincides with feeding time.

### 2.4. Sample Collection and Index Determination

#### 2.4.1. Feed Intake Sampling and Calculations

Feed intake was recorded before feeding in the morning. The cows were allowed to eat freely to ensure that each cow had sufficient feed intake. Surplus was measured weekly for each cow. DMI was calculated based on a 10% residual quantity $\mathrm{DMI}=\left(\mathrm{quantity}\;\mathrm{of}\;\mathrm{feed}\;\mathrm{supplied}-\mathrm{remaining}\;\mathrm{quantity}\right)*\mathrm{dry}\;\mathrm{matter}*\%$. $Feed\;efficiency=\frac{FCM}{DMI}*4\%$.

#### 2.4.2. Milk Sampling and Analysis

The cows were milked three times a day at 8:00, 12:00, and 21:00, respectively. Milk production was recorded weekly. Then, a 50 mL milk sample was collected during weekly milking in the morning, afternoon, and evening and mixed at a 4:3:3 ratio. The milk samples were stored at 4 °C with a preservative (0.05% benzoic acid) and sent to Dairy One Cooperative Inc. (Shanghai, China) for analyzing the protein, fat, lactose, and somatic cell count (SCC). The ULTRASONIC (M316924) Milk Analyzer (Essae-Teraoka, Bengaluru, India) was used to analyze milk components such as lactose and protein. The mixed milk samples were analyzed for milk fat using FTIR and a milk F A profile using GLC, as described by Urrutia and Harvatine.

#### 2.4.3. Blood Collection and Sampling Analysis

On days 28, 56, and 84 of the experiment, at 3 h after the morning feeding, blood samples (10 mL/sample) were collected from the cows’ tail veins using two vacuum collection tubes, one with EDTA and one without (EDTA and non-EDTA, respectively). The non-EDTA tube was left undisturbed for 30 min and centrifuged at 3000 r/min for 15 min at 4 °C. The supernatant was collected for serum preparation and stored at −80 °C before testing. After all serum samples were collected, we analyzed total protein (TP), albumin (ALB), globulin (GLB), total cholesterol (TC), triglycerides (TG), high-density lipoprotein (HDL), low-density lipoprotein (LDL), blood glucose (GLu), total bile acid (TBA), lipase (LPS), and hormone-sensitive lipase (HSL) using commercial kits. These indices were detected using a biochemical analyzer (Mindray, Shenzhen, China; BS-420) and the kits (BioSinoBio-Technology & Science, Bejing, China) mentioned above.

#### 2.4.4. Antioxidant Capacity

Total antioxidant capacity (TAOC) and levels of superoxide dismutase (SOD) and glutathione peroxidase (GSH-Px) in the serum samples were determined by the Beijing Sino-UK Institute of Biological Technology.

#### 2.4.5. Statistical Analysis

The data of the four groups of cows were collected and analyzed. Data are presented as the means and the standard error of the mean (SEM). Significant differences were determined through one-way analysis of variance and Tukey’s multiple comparison tests. Data were statistically analyzed using SPSS Statistics software, version 20.0 (IBM Corp., Armonk, NY, USA). Statistical significance was defined at *p* < 0.05, with highly significant values at *p* < 0.01.

## 3. Results

### 3.1. Effect of BAs on Growth Performance in Mid-Lactation Cows

Table 2 presents the effects of BAs on dairy cow lactation performance. No significant difference was observed between the experimental and control groups from day 1 to day 28 and from day 29 to day 56 (*p* > 0.05). The dry matter intake of the experimental group with 18 g/head/d was significantly higher than that of the control group from day 57 to day 84 (*p* < 0.05), but no significant difference was observed among the experimental groups (*p* > 0.05). During the experimental period, the dry matter intake was higher in the experimental group than in the control group, but the difference was not significant (*p* > 0.05).

The diets supplemented with different BA levels had no significant effect on the average milk yield at 1–28 d, 29–56 d, and 57–84 d (*p* > 0.05), but the average milk yield was higher in the experimental group than in the control group at all stages.

Different BA supplemental levels exhibited no effect on the 4% milk fat-corrected milk volume at 1–28 d, 29–56 d, and 57–84 d, and no significant difference was observed between the experimental and control groups (*p* > 0.05). The 4% milk fat-corrected milk volume was higher in the experimental group than in the control group at all stages.

Different BA supplemental levels exerted no effect on FCR at 1–28 d, 29–56 d, 57–84 d, and the whole trial period, and no significant difference was observed between the experimental and control groups (*p* > 0.05).

No significant difference in FCR was observed between the experimental and control groups (*p* > 0.05).

### 3.2. Effect of BAs on Milk Composition in Mid-Lactation Cows

Table 3 presents the effects of BAs on the milk composition of dairy cows. Different BA contents in diets at 1–28 d, 29–56 d, and 57–84 d exerted no effects on the average milk fat percentage, lactose percentage, and SCC, but no significant differences were observed between the control and experimental groups (*p* > 0.05). The milk protein rate of the experimental groups supplemented with 18 g/head/d was significantly higher than that of the other experimental groups at 1–28 d (*p* < 0.05).

### 3.3. Effect of BAs on Serum Biomarkers in Mid-Lactation Cows

The effects of BAs on serum biochemistry in lactating cows are displayed in Table 4. No significant difference in the serum TP content was observed between the experimental and control groups on the 28th day (*p* > 0.05). On the 84th day, the serum TP content of the experimental groups supplemented with 12 g/head/d and 18 g/head/d was significantly higher than that of the control group (*p* < 0.05).

The serum ALB content did not differ significantly between the experimental and control groups on days 28 and 56 (*p* > 0.05). On the 84th day, the serum ALB content of the experimental groups supplemented with 12 g/head/d and 18 g/head/d was significantly higher than that of the control group (*p* < 0.05).

The average GLB content exhibited no significant difference between the experimental and control groups on the 28th, 56th, and 84th days (*p* > 0.05).

No significant difference was observed in the average TC content between the experimental and control groups on the 28th and 84th days (*p* > 0.05). On the 56th day, the TC content was significantly lower in the experimental group supplemented with 18 g/head/d than in the control group (*p* < 0.05).

No significant difference in the average TG content was observed between the experimental and control groups on the 28th day (*p* > 0.05). On the 56th day, the TG content of the experimental group supplemented with 18 g/head/d was significantly lower than that of the other experimental groups (*p* < 0.05). The TG content of the experimental group supplemented with 6 g/head/d was significantly lower than that of the control group on the 84th day (*p* < 0.05).

No significant difference was observed in the average HDL content between the experimental and control groups on the 28th and 84th days (*p* > 0.05). On day 56, the average HDL content was significantly higher in the control group than in the experimental group supplemented with 18 g/head/d (*p* < 0.05).

The LDL, CLu, and TBA contents were not affected by the different amounts of BAs added to the diet on the 28th, 56th, and 84th days. No significant difference was observed between the experimental and control groups (*p* > 0.05).

The average LPS content was significantly higher in the experimental groups than in the control group on the 28th day (*p* < 0.01). On the 84th day, the average LPS content of the experimental groups was significantly higher than that of the control group (*p* < 0.01).

On the 28th day, the average HSL content of the experimental group supplemented with 18 g/head/d was significantly higher than that of the control group and other experimental groups (*p* < 0.01). The average HSL content on the 56th day was significantly higher in the experimental group supplemented with 18 g/head/d than in the control group and other experimental groups (*p* < 0.01). On the 84th day, the average HSL content of the experimental group supplemented with 18 g/head/d was significantly higher than that of the control group and other experimental groups (*p* < 0.01).

### 3.4. Effect of BAs on Serum Antioxidant Indices in Mid-Lactation Dairy Cows

Table 5 presents the effects of BAs on the serum antioxidant indices of lactating cows. On the 28th day, SOD levels were significantly higher in the experimental group supplemented with 18 g/head/d than in the control group and the experimental group supplemented with 12 g/head/d (*p* < 0.01). The SOD levels in the experimental group supplemented with 18 g/head/d were significantly higher than those in the control group and other experimental groups on the 56th day (*p* < 0.05). The SOD levels on the 84th day were significantly higher in the experimental group supplemented with 18 g/head/d than in the control group and other experimental groups (*p* < 0.01).

The TAOC of the 18 g/head/d group on day 28 and day 56 was significantly higher than that of the control group (*p* < 0.05). On the 84th day, the TAOC of the 6 g/head/d group and 18 g/head/d group was significantly higher than that of the control group (*p* < 0.05).

Compared with the control group, the GSH-Px levels in the 6 g/head/d group and 12 g/head/d group were significantly higher on the 28th day (*p* < 0.05). The GSH-Px levels in the 18 g/head/d group were significantly higher than those in the other experimental groups on the 56th day (*p* < 0.05). On the 84th day, the GSH-Px levels were significantly higher in the 18 g/head/d group than in the other experimental groups (*p* < 0.01).

## 4. Discussion

### Effect of BAs on the Growth Performance of Mid-Lactation Cows

BAs have been reported in animal production to enhance growth performance, improve fat digestion, lower cholesterol levels, and improve intestinal health in livestock [19]. The addition of bile acids to the basal dose is helpful in improving the efficiency of nutrient utilization and the production performance of animals. Studies have reported the addition of BA or bile salts to broiler diets to assess the effectiveness of BA or bile salts in improving growth performance [20,21]. The basal diet supplemented with pig BA can improve the growth performance, growth and development performance, and intestinal development of weaned piglets [22]. However, there are few studies on the effects of BA on the lactation ability and antioxidant capacity of mid-lactating cows. This experiment was conducted to study the effects of BA on the lactation ability, nutrient intake, and antioxidant capacity of dairy cows. The antioxidant capacity of lactating dairy cows may be affected by malnutrition, environmental stress, metabolic load, disease, and stress, resulting in the decline of the antioxidant capacity of dairy cows. In this study, it was found that dietary supplementation of pig BA could improve the antioxidant capacity of dairy cows, which may be the result of enhanced antioxidant enzyme activity, indicating that dietary supplementation of pig BA is beneficial to dairy cows in mid-lactation. Thus, dietary supplementation of pig BA initially had no significant effect on performance, but may play a role in homeostatic mechanisms by affecting serum antioxidant enzyme and antioxidant levels. In addition, BAs have antibacterial properties and are more sensitive to Gram-positive bacteria than Gram-negative bacteria. Antioxidant status may also be improved due to reduced colonization of the gastrointestinal tract by pathogenic bacteria, but this requires specific detailed studies.

Monitoring and managing DMI is critical for dairy health, performance, and feed efficiency [23]. Dry matter intake refers to the amount of dry matter consumed by dairy cows, which directly affects the health and performance of cattle. The milk production and body weight gain of dairy cows are closely related to the amount of dry matter they consume [24]. Adequate dry matter intake helps to improve milk production and maintain good body weight status, thus improving the benefits of farming. In this experiment, dietary supplementation of pig BA increased the dry matter intake of dairy cows, which may be the reason for the increase in milk production.

The liver plays a vital role in lipid and cholesterol metabolism. One of the major functions of BAs in lipid metabolism is lipoprotein synthesis [25]. Many studies have reported that BAs have lipid-lowering functions [26]. BAs downregulate srebp1 and fxr expression and upregulate HSL expression through the receptor fxr, thereby inhibiting the expression of lipid synthesis-related genes and thus inhibiting lipid synthesis. In our study, BA supplementation decreased the TG and TC content associated with higher serum LPL and LPS levels in mid-lactation cows. The four major classes of plasma lipoproteins are chylomicrons, very LDL, LDL, and HDL. In addition to the liver, the plasma transports substantial amounts of plasma LDL and HDL. LDL transports cholesterol esters from the liver to other organs, whereas HDL removes cholesterol from peripheral tissues and transports them to the liver. Then, hepatic cholesterol is converted into BA. In the present study, no differences in TBA levels were observed between the groups, which is consistent with the results of Wang et al. [27], who found that BA supplementation had no effect on endogenous BA synthesis. Wang et al. found that deoxycholic acid, a bile acid, decreases HDL levels in humans [27,28]. In the present study, serum HDL levels decreased with an increase in the BA supplemental levels on day 56, which indicated that BA alters lipid metabolism in mid-lactation cows.

Insulin-like Growth Factor I (IGF-I) plays an important role in animal production. IGF-I is a polypeptide hormone, which is synthesized by the liver and other tissues in animals. Its main role is to promote cell growth, proliferation, and differentiation, and it has an important impact on animal growth, development, and metabolism. Insulin-like growth factors regulate growth and metabolism in animals through the brain neuro–endocrine axis GH/IGF-I, a central hormone for growth regulation [29]. Serum IGF-I levels increased with increasing dietary BA levels, indicating that BAs had a beneficial effect on IGF-I concentrations, thereby improving lactation performance in mid-lactation cows. Insulin-like Growth Factor I (IGF-I) plays an important role in animal production. IGF-I is a polypeptide hormone, which is synthesized by the liver and other tissues in animals [30]. Its main role is to promote cell growth, proliferation, and differentiation, and it has an important impact on animal growth, development, and metabolism [31]. Insulin-like growth factors regulate growth and metabolism in animals through the brain neuro–endocrine axis GH/IGF-I, a central hormone for growth regulation [32]. Serum IGF-I levels increased with increasing dietary BA levels, indicating that BAs had a beneficial effect on IGF-I concentrations, thereby improving lactation performance in mid-lactation cows.

## 5. Conclusions

Adding BAs to the basic diet of dairy cows can improve the lactation performance of mid-lactation cows. The improvement in lactation performance of dairy cows may be the result of increased serum antioxidant capacity and IGF-I. In addition, the addition of BAs alters the composition of milk and lipid metabolism, improving the health status of mid-lactation cows. In conclusion, BAs can be supplemented in the basic diet of dairy cows to improve their lactation performance and health status.

## Figures and Tables

**Table 1:** Ingredients and nutrient composition of the experimental diet (% of DM)**.**

Item	%
Powder mix ^1^	22.73
Expanded soybean meal	2.19
Soybean hulls	2.19
Whole cottonseed	4.39
Alfalfa	3.95
Oats	3.29
Alfalfa silage	6.58
Alfalfa corn	48.25
Molasses	2.19
Yeast	0.07
Fat powder	0.66
Lysine	0.02
Sodium bicarbonate	0.64
Sodium chloride	1.21
Methionine	0.07
Urea	0.18
Vitamin and mineral mix ^2^	1.39
Nutrient composition	
NEL ^3^ (MJ/kg)	6.78
CP	16.15
NDF	33.51
ADF	21.72
Ash	7.12
Ca	0.42
P	0.25
EE	3.75

^1^ Powder mix contained 70% corn and 30% soybean meal. ^2^ Vitamin and mineral mix contained 510 mg/kg Cu, 1380 mg/kg Zn, 2700 mg/kg Mn, 26 mg/kg Se, 170 mg/kg Fe, 20 mg/kg I, 4 mg/kg, 180 kIU/kg vitamin A, 45 kIU/kg vitamin D, and 1400 kIU/kg vitamin E. ^3^ Except for NEL, which is a calculated value, the rest are measured values.

**Table 2:** Effects of dietary addition of BAs on DMI, milk yield, and milk composition in dairy cows.

Item	Day	BA Addition, g/head/d				SEM	*p* Value
		0	6	12	18		
DMI (kg/d)	1–28	25.01	26.61	25.50	26.27	0.26	0.111
	29–56	23.92	24.79	24.56	25.47	0.26	0.225
	57–84	22.42 ^b^	23.42 ^ab^	23.71 ^ab^	24.20 ^a^	0.23	0.041
Milk yield (kg/d)	1–28	36.50	37.87	36.62	38.34	0.48	0.444
	29–56	35.22	36.50	35.27	36.74	0.44	0.492
	57–84	33.48	35.43	34.11	34.39	0.44	0.472
4% FCM (kg/d) ^1^	1–28	33.87	35.83	34.39	34.16	0.82	0.848
	29–56	33.24	34.78	33.84	34.31	0.81	0.923
	57–84	31.01	35.17	32.22	31.96	1.04	0.544
Feed efficiency ^2^	1–28	1.50	1.40	1.47	1.47	0.03	0.681
	29–56	1.49	1.41	1.40	1.41	0.03	0.740
	57–84	1.55	1.49	1.42	1.40	0.03	0.397

^1^ 4% FCM (kg/d) = 0.4 × milk yield (kg/d) + 15 × milk yield (kg/d) × milk fat (%). ^2^ Feed efficiency = 4% FCM/DMI. ^a,b^ Means in the same row with different superscripts differ significantly for treatment effect.

**Table 3:** The effect of supplementing BA on the composition of milk in mid-lactation cows.

Item	Day	BA Addition, g/head/d				SEM	*p* Value
		0	6	12	18		
Milk fat (%)	1–28	3.89	3.92	3.96	3.98	0.04	0.896
	29–56	3.90	3.94	3.96	3.98	0.05	0.939
	57–84	3.82	3.87	3.91	3.89	0.03	0.781
Milk protein (%)	1–28	3.25 ^b^	3.27 ^b^	3.28 ^b^	3.38 ^a^	0.02	0.040
	29–56	3.19	3.26	3.27	3.36	0.02	0.980
	57–84	3.12	3.19	3.18	3.27	0.02	0.132
Milk lactose (%)	1–28	5.07	5.08	5.08	5.09	0.01	0.928
	29–56	5.04	5.06	5.04	5.05	0.02	0.980
	57–84	4.88	4.91	4.89	4.93	0.02	0.782
SCC (×10^3^/mL)	1–28	50.33	48.78	51.63	53.27	2.58	0.939
	29–56	51.64	47.88	50.66	50.89	2.89	0.971
	57–84	52.62	50.98	49.92	50.36	2.94	0.989

^a,b^ Means in the same row with different superscripts differ significantly for treatment effect.

**Table 4:** The effect of supplementing BA on serum routine indicators in mid-lactation cows.

Item	Day	BA Addition, g/head/d				SEM	*p* Value
		0	6	12	18		
TP (g/L)	28	61.91	69.45	67.21	61.13	1.68	0.225
	56	66.99	61.36	60.66	52.22	2.26	0.142
	84	26.52 ^b^	35.69 ^ab^	37.72 ^a^	41.79 ^a^	1.75	0.013
ALB (g/L)	28	31.49	36.05	34.80	31.82	0.86	0.163
	56	35.36	30.47	31.52	27.74	1.07	0.085
	84	14.72 ^b^	18.47 ^ab^	19.52 ^a^	22.90 ^a^	0.82	0.004
GLB (g/L)	28	30.42	36.07	32.40	29.31	1.15	0.170
	56	31.63	30.90	29.15	24.48	1.36	0.247
	84	11.80	17.22	18.21	18.88	1.14	0.109
TC (mmol/L)	28	7.01	8.73	8.07	7.14	0.27	0.069
	56	8.41 ^a^	7.32 ^ab^	7.59 ^ab^	6.01 ^b^	0.29	0.031
	84	5.20	4.32	4.68	4.74	0.19	0.469
TG (mmol/L)	28	0.18	0.20	0.18	0.17	0.01	0.396
	56	0.19 ^a^	0.17 ^b^	0.17 ^b^	0.14 ^c^	0.01	0.048
	84	0.15 ^a^	0.11 ^b^	0.13 ^ab^	0.12 ^ab^	0.01	0.023
HDL (mmol/L)	28	3.88	4.48	4.38	3.91	0.11	0.121
	56	4.60 ^a^	3.84 ^ab^	4.10 ^ab^	3.32 ^b^	0.14	0.011
	84	1.80	2.31	2.51	2.57	0.11	0.060
LDL (mmol/L)	28	1.43	1.74	1.57	1.46	0.05	0.128
	56	1.68	1.58	1.58	1.31	0.06	0.090
	84	1.21	1.09	1.19	1.12	0.04	0.745
Glu (mmol/L)	28	3.19	3.47	3.40	3.06	0.08	0.273
	56	2.99	2.94	2.63	2.68	0.08	0.304
	84	2.33	2.13	2.07	2.15	0.07	0.586
TBA (μmol/L)	28	72.12	55.34	71.90	68.52	4.44	0.506
	56	59.73	44.77	51.82	43.55	3.43	0.321
	84	32.77	28.06	39.89	40.02	2.54	0.271
LPS (U/mL)	28	63.10 ^b^	70.83 ^a^	70.54 ^a^	71.73 ^a^	0.79	<0.001
	56	70.28	69.24	65.39	73.47	1.11	0.076
	84	63.10 ^b^	72.61 ^a^	78.42 ^a^	75.68 ^a^	1.46	0.001
HSL (pg/mL)	28	60.96 ^b^	61.52 ^b^	63.19 ^b^	66.61 ^a^	0.52	<0.001
	56	62.49 ^c^	66.20 ^b^	66.05 ^b^	69.87 ^a^	0.67	0.001
	84	61.11 ^c^	65.26 ^b^	66.21 ^b^	72.45 ^a^	0.75	<0.001

Abbreviation: TP—total protein; ALB—albumin; GLB—globulin; TC—total cholesterol; TG—triglyceride; HDL—high-density lipoprotein; LDL—low-density lipoprotein; Glu—blood glucose; TBA—total bile acid; LPS—lipase; HSL—hormone-sensitive lipase. ^a,b,c^ Means in the same row with different superscripts differ significantly for treatment effect.

**Table 5:** The effect of supplementing BA on the antioxidant capacity of mid-lactation cows.

Item	Day	BA Addition, g/head/d				SEM	*p* Value
		0	6	12	18		
SOD (U/mL)	28	69.03 ^c^	77.22 ^ab^	75.43 ^b^	78.62 ^a^	0.67	<0.001
	56	75.10 ^b^	76.79 ^b^	74.94 ^b^	81.51 ^a^	0.89	0.026
	84	78.07 ^b^	75.61 ^b^	76.32 ^b^	91.62 ^a^	1.26	<0.001
TAOC (U/mL)	28	7.66 ^b^	8.49 ^ab^	8.38 ^ab^	8.90 ^a^	0.16	0.043
	56	7.37 ^b^	8.41 ^ab^	8.19 ^ab^	8.93 ^a^	0.19	0.023
	84	7.49 ^b^	8.83 ^a^	8.64 ^ab^	9.11 ^a^	0.22	0.046
GSH-Px (U/mL)	28	349.49 ^b^	376.53 ^a^	373.98 ^a^	363.83 ^ab^	3.55	0.025
	56	367.10 ^b^	378.58 ^b^	381.73 ^b^	408.75 ^a^	4.95	0.019
	84	385.96 ^b^	393.43 ^b^	388.89 ^b^	419.52 ^a^	3.71	0.003

Abbreviation: SOD—superoxide dismutase; TAOC—total antioxidant capacity; GSH-Px—glutathione peroxidase. ^a,b,c^ Means in the same row with different superscripts differ significantly for treatment effect.

## Data Availability

Data are contained within the article.
